# Use of carbon dioxide for therapeutic decision-making in endoleaks: a case report

**DOI:** 10.1590/1677-5449.200060

**Published:** 2020-11-16

**Authors:** Adalberto Batalha Megale, Cynthia de Almeida Mendes, Marcelo Passos Teivelis, Carolina Brito Faustino, Kauê Polizel Souza, Nelson Wolosker

**Affiliations:** 1 Hospital Israelita Albert Einstein, São Paulo, SP, Brasil.

**Keywords:** endoleak, endovascular procedure, aneurism, carbon dioxide, angiography, endoleak, procedimento endovascular, aneurisma, dióxido de carbono, angiografia

## Abstract

Endovascular aneurysm repair is currently the most frequently treatment modality for infrarenal aortic aneurysms. Endoleaks are the most common cause of reintervention after endovascular aneurysm repair. It is often unclear which type of endoleak is the correct diagnose, making the treatment decision difficult. We report the case of a 72-year-old man with an endoleak two years after endovascular aneurysm repair. Images suggested a type III endoleak, but this was not confirmed by contrast aortography. We proceeded with the investigation using aortography with carbon dioxide and observed a type IA endoleak. This was successfully treated by implantation of a proximal cuff. A review of the literature shows that the role of carbon dioxide in endoleak management is still unclear. We present a case in which carbon dioxide was essential to both diagnosis and therapeutic decision-making in a type IA endoleak.

## Resumo

O tratamento endovascular dos aneurismas de aorta abdominal é atualmente a modalidade de tratamento mais comum. Os *endoleaks* representam a causa mais frequente de reintervenção após o tratamento endovascular. O diagnóstico do tipo de *endoleak* frequentemente é incerto, tornando o tratamento desafiador. Apresentamos o caso de um paciente de 72 anos, com *endoleak* após 2 anos de tratamento endovascular de aneurisma de aorta abdominal. Os exames de imagem pré-operatórios sugeriam um *endoleak* tipo III; entretanto, durante aortografia com contraste iodado, não foi possível identificá-lo. Optamos por realizar aortografia com dióxido de carbono (CO_2_), sendo, então, identificado um *endoleak* tipo IA, que foi tratado com sucesso com o uso de uma extensão (*cuff*) proximal. O papel do CO_2_ no diagnóstico de *endoleaks* ainda não está claro. Relatamos um caso em que o uso do CO_2_ foi essencial para o diagnóstico e para a decisão de tratamento do *endoleak* tipo IA.

## INTRODUCTION

Endovascular repair (EVAR) is now the most frequent treatment modality for infrarenal aortic aneurysms.^1^ It is associated with lower 30-day mortality rates compared to open repair.^2^ EVAR is also correlated with a higher reintervention rate, which can be as high as 20% in the first 5 years.^3^

Endoleaks constitute the main cause of reinterventions.^4^ It is often unclear which type of endoleak is the correct diagnose,^5^ making the treatment decision difficult.

Diagnostic aortography is generally performed with iodinated contrast. However, even with power injectors and high doses of iodinated contrast, there is occasionally still doubt with regard to which type of endoleak is present. In this situation, carbon dioxide (CO_2_) represents an alternative method that can solve the problem.^6^

We conducted a literature review and were unable to find any papers reporting use of CO_2_ as being crucial for the decision on how to treat an endoleak.^7^

We report a case in which an endoleak identified after EVAR was defined as type III by computed tomography angiography (CTA) and contrast-enhanced ultrasound (CEU). However, during the intervention, iodinated contrast aortography did not show presence of any endoleaks, but when aortography with CO_2_ was used, a type IA endoleak was evident. This was successfully treated with placement of a proximal cuff in the aorta.

## CASE REPORT

We describe the case of a 72-year-old male patient with a past medical history of hypertension and atrial fibrillation treated by anticoagulation with warfarin. He originally presented with an infrarenal aortic aneurysm with a maximum diameter of 6.5 cm and a concomitant left common iliac artery aneurysm of 4.9 cm.

The patient was successfully treated by EVAR with Incraft^®^ main body - 30mm x 98mm; ipsilateral branch - 13mm x 140mm; and contralateral branch - 24mm x 100mm (Cordis, Cardinal Health, Dublin, OH, EUA) and embolization of the left internal iliac artery with coils, two years ago.

In accordance with our institutional protocol, the patient underwent CTA 30 days postoperatively, showing a type II endoleak connecting the left iliac artery aneurysm (embolized), inferior mesenteric artery, and two lumbar arteries, with expansion of the aneurysm sac to 7 cm.

Since the patient was under anticoagulation, we first attempted cessation of warfarin for 3 months and clinical follow-up. After that period, CTA showed the same endoleak, but with stability of the aneurysm diameter and less contrast flow in the sac.

After 6 months, a repeat CTA demonstrated stability of the aneurysm diameter.

**Figure 1:**
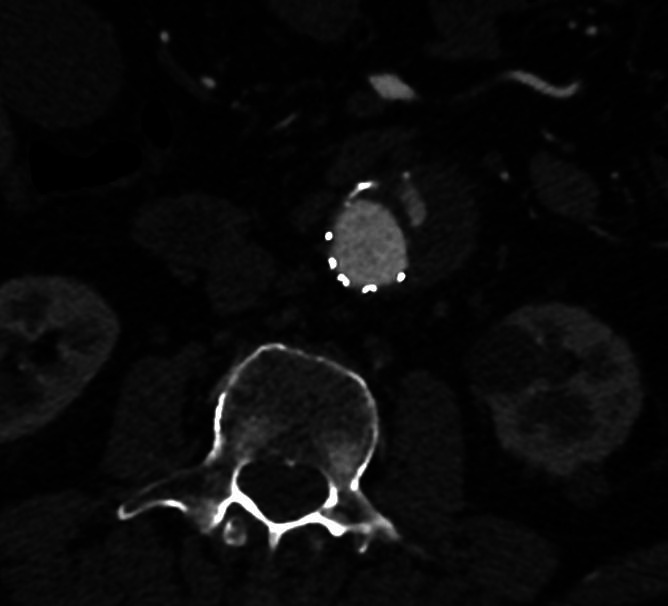
CTA 1 year after the EVAR showing leaking contrast at the anterior face of the main endograft body.

At the 1-year control CTA, a 7.1 cm diameter sac was observed with no remaining type II endoleaks. However, a new contrast area was seen on the anterior surface of the stent graft, which was suspected to be a type III endoleak (Figure 1).

**Figure 2:**
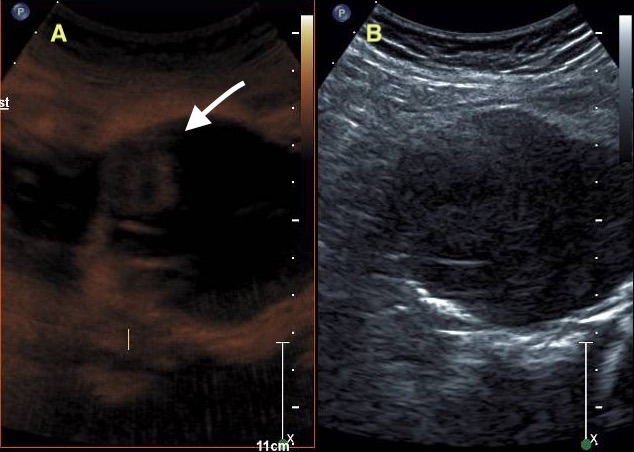
(A) CEU showing endoleak in the anterior face of the endograft; (B) B-mode image of the endograft.

During further investigation, CEU showed a high-flow endoleak on the anterior surface and a possible stent fracture. Also, a low-flow endoleak of the anterior lumbar and the left internal iliac arteries were observed with CEU (Figure 2).

At this point the decision was taken to proceed with endovascular treatment, due to the small change in the aneurysm sac and the suspicion of an endograft fracture.

**Figure 3:**
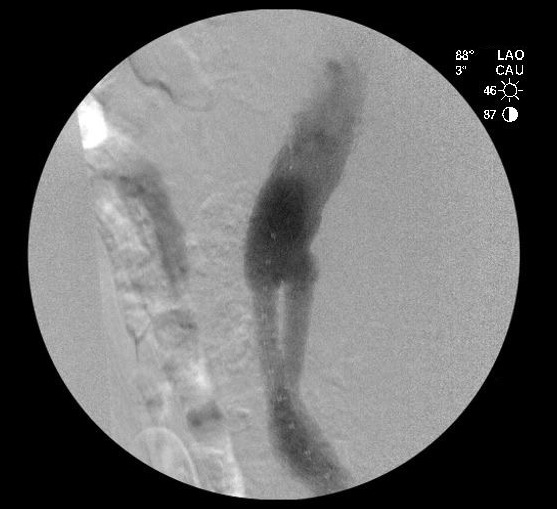
Aortography with iodinated contrast and power injector with no visible endoleak.

During the intervention, aortography was performed with iodinated contrast and a power injector in 30-degree left oblique and 90-degree left oblique incidence angles, and no endoleaks were identified (Figure 3).

Different positions were assessed with the pigtail catheter: next to the suprarenal fixation, to the main body of the endograft, and inside the iliac branches. No contrast flow was seen in the aneurysm sac.

We further performed aortography with CO_2_. The first injection was made manually with a pigtail catheter and 30 ml of CO_2_ in a 60 ml syringe with a 90-degree left oblique incidence angle, but no endoleaks were observed.

**Figure 4:**
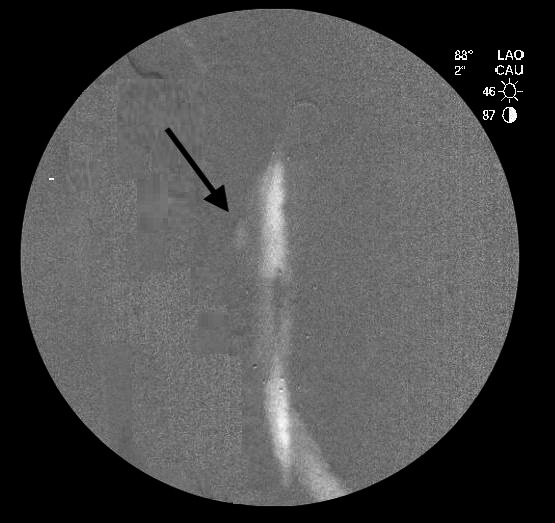
Aortography with CO_2_ and Simmons 1 catheter. An endoleak was identified in the free-flow area (black arrow), arising from the proximal part of the main body.

Finally, we used a Simmons 1 catheter with the same incidence and volume, identifying an endoleak at the anterior surface, originating from the proximal portion of the endograft, which we characterized as a type IA endoleak (Figure 4).

No signs of fracture or contrast leakage from the stent graft could be seen.

After this diagnosis, the patient was successfully treated with a proximal cuff (Incraft® 34 mm x 42 mm). Control aortography with CO_2_ showed no endoleaks.

Thirty days after the intervention, another CTA was performed, showing no contrast in the aneurysm sac.

## DISCUSSION

Endoleaks are the main cause of reintervention after EVAR. When type I endoleaks are identified at the time of the final aortography after EVAR, they are usually considered to justify treatment.^4^ Type II endoleaks are mostly benign. However, in a few cases, they can cause enlargement of the sac and can eventually lead to rupture.^8^ Type III endoleaks are associated with a higher risk of rupture and aneurysm sac enlargement and should always be treated.^9^

Conflicting diagnostic results regarding the type of endoleak are frequent. In a 2019 study by Madigan et al. with 130 patients with type II endoleaks, 22% of the patients who needed treatment had a type I or III endoleak that had been previously misidentified and were only correctly diagnosed during the intervention.^9^

In the case reported here, the patient was followed-up in order to investigate the type II endoleak and, due to sac enlargement and suspicion of a stent graft fracture, a treatment decision was necessary.

During the preoperative investigation, a type III endoleak had been considered, but it was only during the endovascular procedure, after CO_2_ injection, that a type I endoleak was identified.

It is important to remember that both CTA and CEU results suggested a type III endoleak, and only CO_2_ aortography was capable of demonstrating that the endoleak was actually from the proximal landing zone of the endograft (type IA).

It is no novelty that CO_2_ can be used as an alternative contrast for EVAR. In 2011, Criado et al. performed 114 EVAR procedures with CO_2_ and observed 20 endoleaks (two type I, sixteen type II, and two type IV). No additional endoleaks were diagnosed with iodinated contrast. No type I or III endoleaks were identified with CTA after the first month of follow-up.^10^

The comparison between CO_2_ and iodinated contrast for detecting endoleaks is still under debate.

In 2018, Mascoli et al. compared the accuracy of CO_2_ with iodinated contrast and CEU in 21 patients. All type I and III endoleaks were detected with iodinated contrast and CO_2_. Seven type II endoleaks were identified with CO_2_; of these, only one was diagnosed with iodinated contrast and four with CEU; therefore, two of them were only recognized using carbon dioxide.^5^

In 2015, Sueyoshi et al. observed that all type I and type III endoleaks were identified by both methods (iodinated contrast and CO_2_), but CO_2_ was less sensitive than iodinated contrast for detecting type II endoleaks.^2^

An automated CO_2_ injection method for EVAR was presented by Mascoli et al. in 2018. In this study with 31 patients, it was possible to identify all type I and III endoleaks using the automated method. However, they reported that CO_2_ had higher sensitivity for type II endoleaks than iodinated contrast.^11^

Even though CO_2_ was manually injected in this case report, it was only when the gas was used that it was possible to detect the type I endoleak.

One technical point was that the image was only conclusive after injection with an end-hole catheter in the same direction as the blood flow. This technique has been previously described for performing EVAR by Criado et al., in 2011, and by Mendes et al. in 2016.^1^^,^^10^

In 2011, Criado et al. used an end-hole catheter to conduct final angiograms after EVAR.^10^ In 2018, Mascoli et al. injected CO_2_ from the sheath, with the patient in the Trendelenburg position.^6^ This could have enhanced the good sensitivity they achieved with CO_2_ for identifying endoleaks. Sueyoshi et al. did not provide enough information about the method of CO_2_ injection used.^5^

When CO_2_ is used with a pigtail catheter, its low viscosity leads to explosive effect, producing bubbles and resulting in lower quality images. It provides higher quality images when injected with an end-hole catheter.

The patient described in this study was admitted to the operating room with a suspected type III endoleak diagnosed by CEU and CTA. Injection of iodinated contrast excluded this diagnosis, but did not show the location of the endoleak that had been shown in previous images.

Use of CO_2_ permitted detection of the type IA endoleak. This was probably because it was a small endoleak, hence the slow enlargement of the aneurysm sac. Once the endoleak had been diagnosed, it was possible to treat it using a proximal cuff to repair the problem.

## CONCLUSION

Treatment of endoleaks after EVAR represents a challenge. It is important to use multiple diagnostic and therapeutic methods for proper management.

Angiography with CO_2_ constitutes a supplementary method to conventional angiography with iodinated contrast. However, further studies to establish the real accuracy of this method would be welcomed.
